# Neuron-specific transcriptomic dysregulation in methotrexate-induced cognitive impairment revealed by SnRNA-seq

**DOI:** 10.1097/JS9.0000000000003519

**Published:** 2025-09-23

**Authors:** Jishi Ye, Yu Ding, Ruolan Wu, Huang Ding, Juan Ren, Zhongyuan Xia, Jingli Chen, Shuang Xie, Yifan Jia

**Affiliations:** a Department of Pain, Renmin Hospital of Wuhan University, Wuhan, Hubei, People’s Republic of China; b Department of Anesthesiology, Renmin Hospital of Wuhan University, Wuhan, Hubei, People’s Republic of China; c Department of Anesthesiology, The Central Hospital of Wuhan, Tongji Medical College, Huazhong University of Science and Technology, Wuhan, Hubei, China; d Department of Respiratory Medicine, General Hospital of Central Theater Command, Wuhan, China

**Keywords:** cognitive impairment, hippocampus, methotrexate, neurons, single-nucleus RNA sequencing

## Abstract

**Background:**

Methotrexate (MTX) is a widely used chemotherapy drug, but its neurotoxicity can lead to cognitive impairments, particularly through effects on hippocampal function. Nevertheless, the underlying molecular mechanisms are not fully understood. Deciphering MTX-induced cognitive impairment-linked molecular mechanisms in cells of the hippocampus could uncover novel therapeutic targets.

**Methods:**

In this study, we established a mouse model of cognitive impairment induced by the chemotherapy drug MTX. We applied single-nucleus RNA sequencing (snRNA-seq) to analyze the transcriptomic alterations in hippocampal cells of mice following MTX treatment, with a focus on neuron-specific gene expression changes.

**Results:**

MTX chemotherapy led to a decrease in excitatory neurons but an increase in inhibitory neurons, altering the excitatory–inhibitory balance of neural networks and thus mediate cognitive dysfunction. Furthermore, MTX significantly disrupted the transcriptional regulatory network and potential trajectory of GABAergic neurons. It enhanced the Nrg1–Erbb4 pathway while attenuating the Nrxn3–Lrrtm4 pathway, destabilizing trans-synaptic signaling and causing abnormalities in excitatory and inhibitory synaptic functions. These disruptions may ultimately lead to neural network imbalance and cognitive dysfunction.

**Conclusion:**

This study highlights the specific effects of MTX chemotherapy on hippocampal cellular function and provides valuable insights into the molecular mechanisms underlying cognitive deficits and potential therapeutic targets.

**HIGHLIGHTS**

- MTX significantly disrupted the transcriptional regulatory network of GABAergic neurons and weakened cell-to-cell communication.
- This is the first use of single-cell sequencing to explore potential pathogenic mechanisms of CICI.
- Notably, alterations in synaptic transmission pathways, such as Nrg1–Erbb4 and Nrxn3–Lrrtm4, were identified as key contributors to excitatory–inhibitory imbalance and cognitive dysfunction.

## Introduction

Many cancer patients who receive chemotherapy develop chemotherapy-induced cognitive impairment (CICI) during or after treatment, often called “chemo-brain,” which affects patients’ quality of life and imposes a significant burden on healthcare systems worldwide^[1,2]^. There is mounting evidence that CICI involves impairment of learning, memory, attention, and executive function. Its incidence is estimated to affect up to 75% of patients treated with chemotherapy, and approximately 17–35% suffer long-term effects^[2]^. Methotrexate (MTX) is a widely used antimetabolite chemotherapeutic agent, particularly in the treatment of leukemia, lymphoma, breast cancer, and other malignancies^[3–5]^. It functions primarily by inhibiting dihydrofolate reductase (DHFR), thereby disrupting folate metabolism, which is essential for DNA synthesis and cell proliferation. While effective in targeting rapidly dividing cancer cells, MTX is also associated with significant off-target effects, including neurotoxicity^[6,7]^.

Clinically, MTX-induced neurotoxicity manifests in both acute and chronic forms. Acute effects can include encephalopathy, seizures, and stroke-like symptoms, whereas chronic exposure is strongly linked to cognitive deficits, often referred to as “chemobrain.” These deficits involve impairments in memory, attention, processing speed, and executive function. Mechanistically, MTX has been shown to disrupt neurotransmitter balance, increase oxidative stress, induce neuroinflammation, and impair neurogenesis^[8,9]^.

MTX and other chemotherapy agents can cause neurotoxicity in multiple brain regions in both patients and experimental animals. The hippocampus is particularly affected, showing neuronal loss, reduced volume, and impaired neurogenesis, leading to memory and learning deficits^[8,10]^. The prefrontal cortex also suffers damage, resulting in executive function and attention impairments[11]. The basal ganglia may be involved, affecting motor control, motivation, and emotional regulation[12], while the cerebellum experiences oxidative stress and synaptic changes, leading to motor and balance issues[13]. Although chemotherapy affects various brain regions, the hippocampus is a primary focus due to its key role in cognition and its high susceptibility to neurotoxicity.

Therefore, a deeper understanding of CICI, especially the hippocampal pathogenic mechanisms involved in MTX-induced CICI, is essential for developing effective prevention strategies. Utilizing various advanced neuroimaging techniques, preclinical animal studies have reported a variety of putative mechanisms, including direct neurotoxic effects, blood–brain barrier disruption, reduced hippocampal neurogenesis, altered neuronal proliferation and apoptosis, white matter abnormalities, secondary inflammatory response, increased oxidative stress, and changes in cerebral blood flow^[1,14–16]^. Additionally, neurochemical changes manifesting as alterations in neurotransmitter levels, metabolic changes, and hormone-driven changes are other potential underlying mechanisms.

Some studies from single-nucleus RNA sequencing (snRNA-seq) datasets revealed profound changes in the transcriptome of vascular, glial, and neuronal cells in different nervous system tumors, providing useful links to the pathogenesis of nervous system tumors^[17–19]^. The hippocampus plays a crucial role in various cognitive functions and exhibits extensive cellular heterogeneity. Identifying cell type-specific gene expression profiles in the hippocampus can enhance our understanding of cognitive functions. The snRNA-seq method was also used to characterize hippocampal changes in mice with central nervous system disease, indicating that capturing precise transcriptional changes during neuroinflammation at the single-cell level is beneficial in exploring disease mechanisms and facilitating drug discovery^[20–22]^.

The effects of chemotherapy drugs on various types of brain cells, particularly the hippocampus, have not been fully explored. In addition, it is unclear whether cognitive decline in patients treated with chemotherapy drugs is related to the destruction of brain cell populations. Therefore, this study aims to explore the specific gene expression profiles of different cell types in the hippocampus of mice treated with MTX using snRNA-seq, with the goal of elucidating the differential effects of MTX treatment on various cell types and potential molecular mechanisms. According to the TITAN criteria, artificial intelligence (AI) was employed exclusively for language polishing and summarization, with the sole purpose of improving the readability, grammar, and structural clarity of the text[23].

## Methods

### Establishment of the CICI mice model

**Figure 1.**
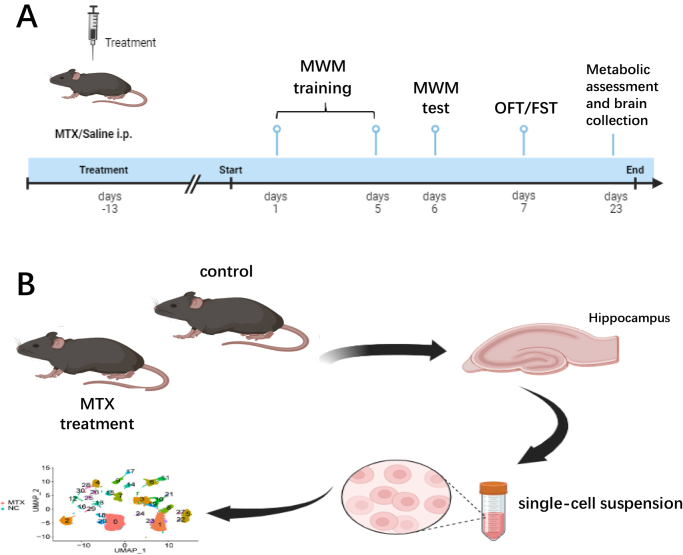
**Experiment-related flowchart.** (A) Schematic detailing study design and timeline. (B) Isolation of mouse hippocampal tissue and single-cell sequencing.

Ethical approval: The Experimental Animal Welfare Ethics Subcommittee of the Biomedical Ethics Committee gave ethical approval for the study. The work has been reported in accordance with the ARRIVE guidelines (Animals in Research: Reporting In Vivo Experiments)[24]. The 7-week-old male C57BL/6**J** mice were obtained from the Hubei Provincial Center for Disease Control and Prevention and maintained under standard conditions (12 h/12 h light–dark cycle, 25°C, 60± 10% humidity, free access to food and water). After a 1-week adaptation period, the mice were randomly divided into two groups (*n* =30 per group): the control group and the methotrexate treatment group. Mice in the MTX treatment group were intraperitoneally injected with MTX (100 mg/kg, dissolved in PBS) every 5 days for a total of three times. The experimental workflow is illustrated in Figure 1. To minimize bias, all behavioral assessments, histological analyses, and transcriptomic data processing were performed by investigators blinded to the treatment groups. Group allocation codes were concealed until the completion of data collection and primary analyses.

### Animal behavior tests

#### Open field test

The open field test (OFT) served to evaluate the locomotor activity (total distance traveled) and anxiety levels (time spent in the central area) of rodents in a novel environment and was conducted using a method similar to that reported previously**^[25,26]^,** with a minor modification. A non-enclosed box with dimensions of 50 cm × 50 cm × 40 cm was used. The mice were placed in the center of an open field and allowed to explore for 5 minutes in a quiet and dim environment. Each animal’s behavior was tracked using behavior-recording software to measure the total distance traveled, time spent in the center, and freezing behavior. Prior to testing, the test chamber was cleaned thoroughly to remove previous odors and excretions using 75% ethanol.

#### Morris water maze

Spatial learning and memory abilities were analyzed in the Morris water maze (MWM). The MWM consists of a circular stainless-steel tank with a 200-cm diameter, filled with water-dyed white using liquid tempera paint to make it opaque. A platform was submerged 1 cm beneath the water’s surface in one of the quadrants. Mice were placed at the designated start point, facing the tank wall, and released at water level. If the mouse does not find the platform within 60 seconds, it is gently placed on the platform for 15 seconds before being removed. The process was repeated four times per day, with the mouse starting from a different location each time. On day 6, a probe test was conducted, where the submerged platform is removed, and the mouse is allowed to swim freely for 60 seconds. Escape latency and time spent in the target quadrant were recorded. Behavioral data from the training phase were analyzed using repeated-measures ANOVA, while the probe test data were evaluated with one-way ANOVA. Visual cue test results were assessed with a two-tailed unpaired *t*-test or repeated-measures ANOVA. All behavioral data were processed using GraphPad Prism 7 software.

#### The forced swim test

In this study, the forced swim test (FST) was conducted following the previous articles**^[27,28]^.** Mice were placed in an acrylic cylinder filled with water to a height of 20 cm for a duration of 10 minutes. The water temperature was maintained at 24 ± 1°C to prevent hypothermia. The immobility time, defined as the period during which the mouse remained still except for minimal movements to keep its head above water, was recorded during the last 4 minutes of the trial.

#### Quantitative real-time PCR (qRT-PCR)

Total RNA was extracted from hippocampal tissues of MTX-treated and control mice using TRIzol reagent (Invitrogen, USA) according to the manufacturer’s instructions. cDNA was synthesized using a reverse transcription kit (Takara, Japan). Quantitative real-time PCR was performed on a QuantStudio 6 Flex system (Applied Biosystems, USA) using SYBR Green Master Mix (Takara, Japan). Relative mRNA expression levels of Nrg1, ErbB4, Nrxn3, and Lrrtm4 were normalized to Gapdh using the 2^−ΔΔCt^ method. All reactions were run in triplicate.

#### Western blot

Hippocampal tissues from MTX-treated and control mice were homogenized in RIPA lysis buffer containing protease and phosphatase inhibitors. Protein concentrations were measured by the BCA assay (Thermo Scientific, USA). Equal amounts of protein were separated by SDS–PAGE and transferred to PVDF membranes. Membranes were blocked with 5% BSA and incubated overnight at 4°C with primary antibodies against IL-1β and TNF-α (Abcam, USA). After washing, membranes were incubated with HRP-conjugated secondary antibodies, and signals were detected using an ECL kit (Bio-Rad, USA).

#### Immunofluorescence staining

Brain sections (20 μm) containing hippocampal regions were fixed in 4% paraformaldehyde, blocked with 5% normal goat serum, and incubated with primary antibodies against Nrg1, ErbB4, Nrxn3, and Lrrtm4 (Abcam, USA) overnight at 4°C. After washing, sections were incubated with Alexa Fluor-conjugated secondary antibodies and counterstained with DAPI. Images were acquired using a confocal microscope (Leica, USA).

#### Tissue dissociation

For snRNA-seq, three male mice from the control group and three from the MTX group were euthanized on day 23. The entire hippocampus was dissected and dissociated using a papain dissociation system (Worthington, Lakewood, NJ, USA). The hippocampal samples were digested with papain (20 U/mL) for 45 minutes at 37°C. After digestion, the cells were resuspended in PBS containing 0.04% wt/vol BSA. Trypan Blue (Sigma-Aldrich, Missouri, USA) assessed cell counts and viability. A total of 100 mg of tissue homogenate was collected, nuclei were extracted according to the instructions of the Boyou Cell Nucleus Isolation Kit (SHBIO, 52009-10, China), and the nuclei were fixed with 100% methanol, and the nuclei were counted was evaluated using a fluorescence cell analyzer [Countstar Rigel S2 (Wuhan)/Mira FL (Northwest China), ALIT Life Science, China] and an inverted microscope for cell nuclear morphology evaluation.

### Processing and quality control of hippocampal snRNA-seq data

The UMI count matrix was converted into the Seurat R package[29] (version 4.3.0). Cells with UMI numbers <1000 or detected genes < 500, or with over 5% mitochondrial-derived UMI counts were considered low-quality cells (*n* = 18 814) and were removed. After filtering, 65 665 high-quality nuclei from three MTX mice and three normal mice hippocampal tissue samples were obtained. Genes detected in less than five cells were removed for downstream analyses.

### Normalization, clustering, and data visualization

After quality control, the UMI count matrix was log normalized. Then, the top 2000 variable genes were used to create potential Anchors with the FindIntegrationAnchors function of Seurat. Subsequently, the IntegrateData function was used to integrate data. To reduce the dimensionality of the snRNA-Seq dataset, principal component analysis (PCA) was performed on the integrated data matrix. With the Elbowplot function of Seurat, the top 50 PCs were used to perform the downstream analysis. The main cell clusters were identified with the FindClusters function offered by Seurat, with resolution set at the default value (res = 0.4). Finally, cells were clustered into 8major cell types. And visualized with tSNE or UMAP plots. To identify the cell type for each cluster, we detected gene markers for each cell cluster using the “FindAllMarkers” function in the Seurat package (v4.3.0) on a natural log scale was at least 0.5 and the difference of percent of detected cells was at least 0.25 and adjusted *P*-value was less than 0.05. We then annotated cell types using ScType tools[30] with expanded previously published marker genes of mice hippocampus.

The raw sequencing data from the snRNA-seq were processed using CellRanger software (10x Genomics v.7.0.0), and reads were mapped to the mouse reference genome (GRCm39, Ensembl version M27) with default options to generate unique molecular identifier (UMI) matrices. The CellRanger outputs were converted to Seurat objects by the Read10X function from the Seurat package (version 4.3.0), followed by cell-level quality control procedure. For each sample, nuclei with UMI numbers < 1000 with detected genes < 500, or with over 5% mitochondrial-derived UMI counts were considered low-quality cells and were removed.

### Differential gene expression analysis

Differentially expressed genes (DEGs) were determined with the FindMarkers/FindAllMarkers function from the Seurat package (one-tailed Wilcoxon rank sum test, values adjusted for multiple testing using the Bonferroni correction). For computing DEGs, all genes were probed so that the expression difference on a natural log scale was at least 0.5 the difference in the percent of detected cells was at least 0.15, and adjusted *P*-value was less than 0.05.

### Transcription factor regulatory network analysis (SCENIC)

The modules of TFs were identified by the SCENIC[31] Python workflow (version 0.11.2) using default parameters (http://scenic.aertslab.org). A TF list was used from the resources of pySCENIC (https://github.com/aertslab/pySCENIC/tree/master/resources). Activated TFs were identified in the AUC matrix, and differentially activated TFs were selected using R package limma[32] based on the fold change (logFC ≥ 0.3 or ≤−0.3) and false discovery rate (FDR ≤ 0.05). To identify cluster-specific regulons (especially for analyses with many cell types, where some regulons are common to multiple of them), we used the Regulon Specificity Score (RSS)[33]. Networks of the modules with TFs and their target genes were visualized by Cytoscape (v3.9.1) (https://cytoscape.org/).

### Cell–cell communication analysis

Cell–cell interactions based on the expression of known ligand–receptor (L–R) pairs in different cell types were inferred using CellChat^[34,35]^ (v2.1.1). To identify potential cell–cell communication networks perturbed or induced in mice hippocampus, we followed the official workflow and, loaded the normalized counts into CellChat, and applied the preprocessing functions identify Over Expressed Genes, identify Over Expressed Interactions and projectData with standard parameters set. As database, we selected the Secreted Signalling pathways and used the precompiled human protein–protein interactions as a priori network information. For the main analyses the core functions compute CommunProb, compute CommunProbPathway and aggregateNet were applied using standard parameters and fixed randomization seeds. Finally, to determine the senders and receivers in the network, the function net Analysis_signallingRole was applied on the netP data slot.

### Functional enrichment analysis

To sort out functional categories of genes, Gene Ontology (GO) terms and KEGG pathways were identified using KOBAS 2.0[36]. Hypergeometric test and Benjamini-Hochberg FDR controlling procedure were used to define the enrichment of each term.

### Pseudotime analysis

Monocle2 (v2.26.0)[36] was performed on neuronal cells to uncover the pseudotime trajectory. Dimensionality reduction and trajectory reconstruction were performed using the standard workflow and default parameters. A single-celled trajectory often includes branch. These branches arise because cells execute different gene expression programs. During development, branching occurs in trajectories as cells make fate choices: one developmental lineage advances along one path, while another gives rise to a second path. Monocle contains extensive functionality to analyze these branching events. BEAM (Branched expression analysis modeling) is a kind of statistical method, used to investigate how gene regulation influences branching events.

### Other statistical analysis

Gene set variation analysis (GSVA) was performed using “GSVA (1.46.0)”^[37]^ package. limma analysis of GSVA score was performed using “limma (3.54.2)” package. AddModuleScore[29] function was used to analyze different scores of gene sets selected from MSigDB (https://www.gsea-msigdb.org/gsea/msigdb/mouse/genesets.jsp). The propeller function takes a SingleCell Experiment or Seurat object as input, extracts the relevant cell information, and tests whether the cell type proportions are statistically significantly different between experimental conditions/groups[38].

## Results

### MTX-treated mice showed altered cognitive and learning function

**Figure 2.**
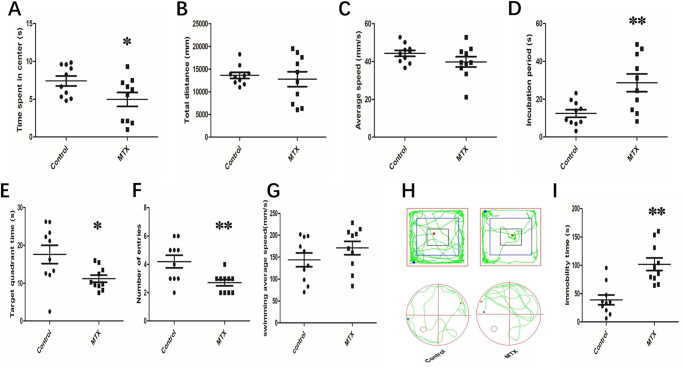
**MTX treatment-induced cognitive behavior injury in mice.** (A) Time spent at the central zone by mice in OFTs. (B) Total distance traveled in the central zone by mice in OFTs. (C) The average speed of mice in OFTs. (D) The incubation period of mice was studied in the Morris water maze (MWM) experiment with a visual platform. (E) The time spent in the target quadrant in the MWM test. (F) The number of entries in the target quadrant of the MWM. (G) The average swimming speed of mice was studied in the MWM. (H) Representative trajectories of each group in MWM (up) and OFT tests (down). (I) The immobility time of mice in FST on day 7.

In the OFT, mice in the MTX group spent less time in the center (Fig. 2A; *P* < 0.05) and entered the center less frequently (Fig. 2H, upper panel) compared with the control group. However, there were no significant differences in total distance traveled and speed between these two groups (Fig. 2B, *P* > 0.05; Fig. 2C, *P* > 0.05).

All data are presented as the mean ± SEM, *n* = 10 for each group. *P< 0.05, **P< 0.01 vs. Control group.

For the MWM tests, in the training test, the escape latency of the MTX group was significantly longer than that of the control group (Fig. 2D; *P* < 0.05). In the spatial probe trial test, we found that the average speed of mice in the MTX group was slower during the test, and the number of times they crossed the original platform area was significantly lower than that of the Control group (Fig. 2F; *P* < 0.05). In addition, we found that mice in the MTX group spent less time in the target quadrant in the MWM test (Fig. 2E; *P* < 0.05). To control for potential motor impairments, swimming speed was also recorded during the MWM task. No significant differences were observed between the MTX and control groups (Fig. 2G, *P* > 0.05).

Meanwhile, we also performed the forced swim test in both groups. The results revealed that the mean immobility time of the control group was significantly higher (Fig. 2I; *P* < 0.05) compared to that of the MTX group. All these findings suggest that MTX induces impairment of learning and memory function in mice.

### snRNA-seq analysis identifies distinct cell types in the hippocampus of MTX-treated and normal mice

**Figure 3.**
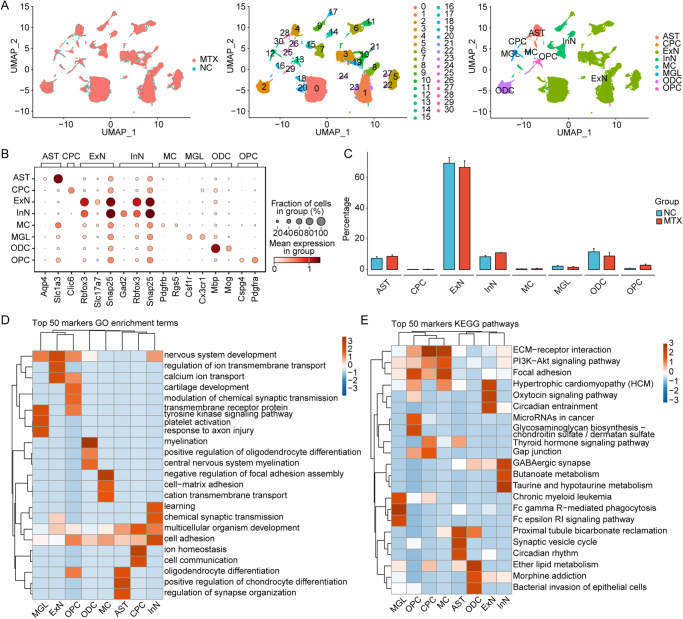
**SnRNA-seq analysis identifies distinct cell types in MTX and normal mice hippocampus.** (A) After quality filtering, normalization, and dimensionality reduction of single-cell data using Seurat, a UMAP clustering map was generated. The left panel displays the groupings, the middle panel shows the clusters and the right panel represents the cell types. (B) Cell type annotations were performed based on the marker genes reported in the literature. (C) The proportions of different cell types within the sample groups are presented. (D) GO functional enrichment analysis of the top 50 marker genes for different cell types, with the top 3 GO-BP pathways displayed for each cell type. (E) KEGG pathway enrichment analysis of the top 50 marker genes for different cell types, with the top 3 KEGG pathways shown for each cell type.

To explore the transcriptomic profile of specific cells in the hippocampus following MTX treatment, we performed snRNA-seq on hippocampal tissues from three MTX-treated mice and three normal mice. After data quality control and removal of doublets, we retained 65 665 nuclei from the 6 samples for subsequent analysis. Through unsupervised clustering analysis and UMAP visualization, a total of 31 cell clusters (C0–30) were initially identified. Subsequently, these cell clusters were annotated based on previously reported literature[39] and some known cell marker genes, leading to the identification of eight main cell types, which included excitatory neurons (ExN), inhibitory neurons (InN), astrocytes (AST), choroid plexus cells (CPC), oligodendrocytes (ODC), oligodendrocyte precursor cells (OPC), microglia (MGL), and mural cells (MC, that is pericytes) (Fig. 3A and B). ExN and InN constitute the vast majority of cells and they broadly express the neuron-specific marker genes Rbfox3 and Snap25. In addition, clusters C7, 9, 14, 15, and 16 were identified as inhibitory neuron clusters due to their high expression of Gad2, whereas other neuron clusters exhibiting high expression of Slc17a7 were classified as excitatory neuron clusters (Supplementary Digital Content Figure S1A, available at: http://links.lww.com/JS9/F180). Subsequently, we presented the differences in cell proportions of various cell types between the MTX group and the normal control (NC) group, revealing that MTX increased the abundance of InN while reducing the number of ExN cells, potentially diminishing the activity intensity of downstream neurons (Fig. 3C). Furthermore, MTX also increased the abundance of OPC and decreased the quantity of ODC.

OPCs not only possess the ability to differentiate into ODCs but are also the only type of glial cells capable of establishing direct synaptic connections with neurons and receiving synaptic inputs from them. MTX-induced OPC activation may modulate inhibitory input to interneurons via synaptic release of GABA, thereby affecting the excitation–inhibition balance within neural networks.

GO functional enrichment analysis of the top 50 feature markers revealed that ExN are primarily involved in neurological system development and transmembrane ion transport, whereas the functions of InN are mainly related to learning and synaptic transmission (Fig. 3D). Although the quantity of InN is far lower than that of ExN, the diversity of InN forms the basis for the brain’s ability to perform complex and refined functions. KEGG pathway enrichment results highlight the importance of InN in GABAergic synapses (Fig. 3E). As a major type of inhibitory synapse, GABAergic synapses are primarily mediated by the neurotransmitter GABA, which can decrease neuronal excitability. Therefore, InN plays a crucial regulatory role in maintaining the excitation–inhibition balance.

In addition, OPC have also been found to be closely associated with the regulation of synaptic transmission (Fig. 3D), further confirming their potential crucial role in the excitation–inhibition imbalance mediated by MTX. Through the analysis of differential gene expression profiles among cell types, we discovered that MTX leads to significant differential expression of numerous genes within OPC (Supplementary Digital Content Figure S1B, available at: http://links.lww.com/JS9/F180). The upregulated genes are primarily involved in processes related to chemical synaptic transmission, neurological system development, differentiation of ODC, and cell adhesion, while the downregulated genes are mainly associated with glutamate receptor signaling, calcium ion transport, and axon guidance (Supplementary Digital Content Figure S1C, available at: http://links.lww.com/JS9/F180).

### Identification of neuron cell subsets

**Figure 4.**
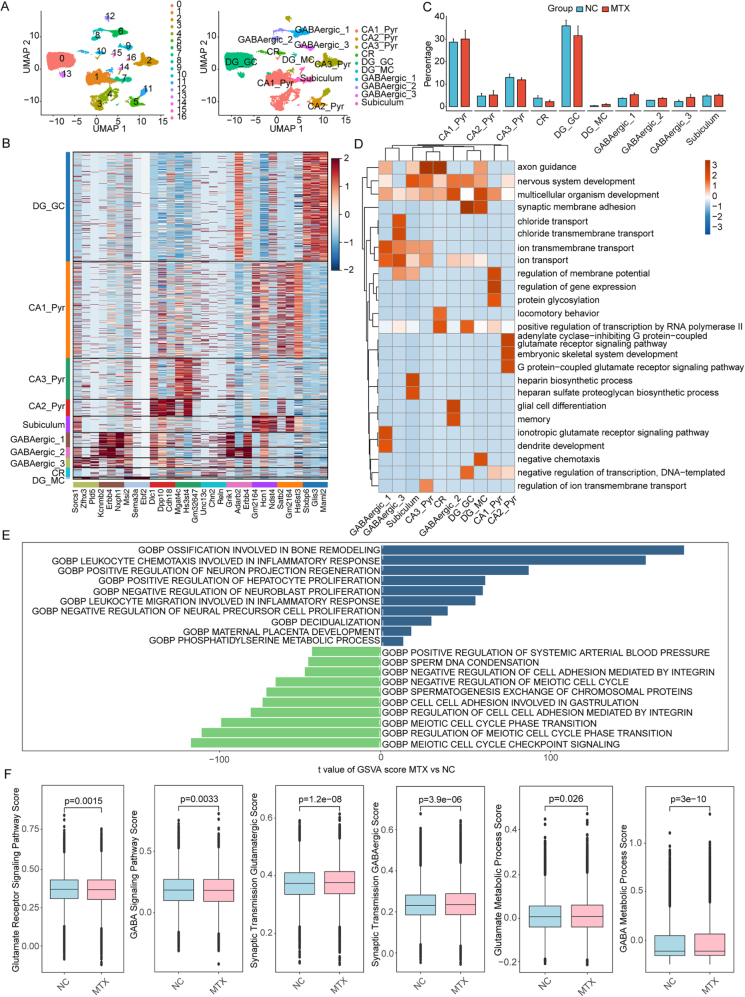
**Identification of neuron cell subsets.** (A) Unsupervised clustering of all extracted neuronal cells, with the UMAP plot showing 17 clusters (left) and 10 major neuronal cell subtypes (right). (B) Heatmap showing the relative expression levels of the top 3 characteristic genes between different neuronal cell subtypes. (C) Bar plot showing the proportions of different neuronal cell subtypes across various sample groups. (D) Heatmap showing GO enrichment pathways for the top 50 marker genes in different neuronal cell subtypes. (E) Diverging bar plot showing the top 10 upregulated/downregulated GO-BP pathways by GSVA scores in neuronal cells. (F) Box plot showing inter-group differences in selected target gene sets in neuronal cells.

To further understand the subtypes of neuronal cells, we isolated all neurons (ExN and InN) and performed secondary clustering, resulting in 17 clusters. Based on cell marker genes reported in the literature^[40,41]^, these clusters were further annotated into 10 major cell subpopulations, corresponding to Dentate gyrus granule cells (DG_GC) and Dentate gyrus mossy cells (DG_MC) of the dentate gyrus, GABAergic neurons, pyramidal neurons in the cornu ammonis (CA_Pyr), subiculum, and Cajal–Retzius (CR) cells (Fig. 4A). The GABAergic neurons and CA regions contain different cell subtypes with distinct physiological functions. The GABAergic neurons were further classified into three types based on the cluster-specific expression of characteristic markers: GABAergic_1 expressing Sst, GABAergic_2 expressing Vip or Cnr1, and GABAergic_3 expressing Pax6 (Supplementary Digital Content Figure S2A and B, available at: http://links.lww.com/JS9/F180). The CA region (broadly expressing Sv2b)was further divided into CA1 (Satb2), CA2 (Pcp4), and CA3 (Spock1). Consistent with previous studies on the hippocampus[42], we similarly found that CA2, in addition to expressing the specific marker Pcp4, also expresses Pfkp, which is highly enriched in CA3, but lacks the CA1 markers Satb2 and Tyro3, revealing its distinct characteristics from CA1 or CA3. This highlights the accuracy of our cell annotation (Supplementary Digital Content Figure S2C, available at: http://links.lww.com/JS9/F180). In addition to these markers associated with cell identity, we also identified high expression of some specific genes among different neuronal cells (Fig. 4B), which may play a role in determining the distinct physiological functions of these cells. Analysis of the proportions of various neuronal cells revealed that the percentage of DG_GC cells decreased in the MTX group, while DG_MC cells increased (Fig. 4C). Additionally, we observed changes in pyramidal neurons, with MTX potentially increasing the proportion of CA1 and CA2 cells while decreasing CA3 cell proportions (Fig. 4C). Additionally, GO functional enrichment analysis of marker genes from each cell type indicated that cells in the DG region are primarily involved in synaptic membrane adhesion processes, while CA3 cells are related to axon guidance. For the three distinct GABAergic neuron clusters, their corresponding GO functions also showed significant differences. GABAergic_1 is potentially associated with the ionotropic glutamate receptor signaling pathway, GABAergic_2 is important for glial cell differentiation and memory, and GABAergic_3 may mediate chloride ion transport (Fig. 4D). Furthermore, GSVA analysis was performed on neuronal cells to characterize the functional pathways significantly related to MTX treatment. We found that MTX significantly upregulated leukocyte-mediated inflammatory responses and negatively regulated neuronal cell proliferation while downregulating integrin-mediated cell adhesion processes (Fig. 4E). This suggests that MTX treatment may exacerbate neuroinflammatory responses in mice and inhibit neuronal regeneration. Additionally, the downregulation of integrin-mediated cell adhesion could lead to reduced synaptic plasticity, thereby impeding synaptic signal transmission. We further assessed the potential effects of MTX on neurotransmitter synaptic transmission in neurons by scoring the activity of relevant gene sets. The results showed that the major excitatory neurotransmitter glutamate receptor signaling pathway was significantly downregulated in the MTX group, while the inhibitory neurotransmitter GABA signaling pathway was significantly upregulated. Additionally, there were notable changes in glutamatergic and GABAergic synaptic transmission and metabolic processes between the MTX and NC groups (Fig. 4F). This indicates that MTX may disrupt the synthesis and release of excitatory and inhibitory neurotransmitters, thereby indirectly affecting receptor binding and signal transduction.

### Identification of cell-specific transcription factors

**Figure 5.**
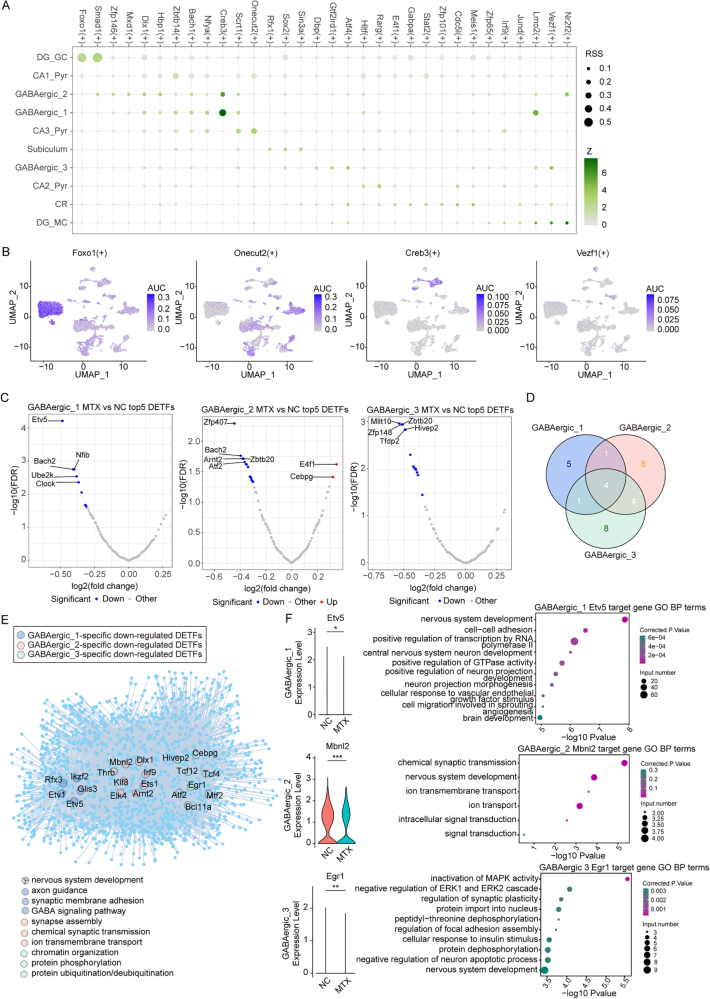
**Identification of cell-specific transcription factors.** (A) Specificity scores of regulators in each neuronal cell subtype. (B) Specific regulons activated in specific subtypes. (C) Volcano plot showing differentially expressed TFs in three GABAergic cell types. (D) Venn diagram showing the overlap of significantly downregulated TFs across three GABAergic cell types. (E) Network diagram showing TF-target relationships specifically downregulated in different GABAergic cell types. (F) Differential expression of selected TFs and GO enrichment results of target genes in different GABAergic cell types.

Synaptic homeostatic regulation ensures the precision of signal transmission between neurons. Using the SCENIC tool, we identified cell-specific regulons in each neuronal subpopulation. These regulons include transcription factors (TFs) and their target genes, which play crucial roles in neuronal development, differentiation, and functional maintenance. By calculating regulon specificity scores (RSS), we discovered that different neuronal subpopulations contain distinctively expressed regulons, indicating transcriptional diversity and specificity among neuronal subpopulations (Fig. 5A). Next, we focused on DG_GC, CA3_Pyr, and GABAergic cells, which were not only previously implicated in potential methotrexate (MTX) treatment effects but also demonstrated specificity with certain regulons, such as Foxo1(+) in DG_GC, Onecut2(+) in CA3_Pyr, Creb3(+) in GABAergic_1 and 2, and Vezf1(+) in GABAergic_3 (Fig. 5B and Supplementary Digital Content Figure S3A, available at: http://links.lww.com/JS9/F180). To further determine whether MTX mediates dysregulation of transcriptional regulatory networks in these cells, we analyzed significantly differentially expressed TFs. Notably, there were almost no significant changes in TF expression detected in excitatory neurons, including DG_GC and CA3_Pyr, whereas we observed extensive downregulation of TFs in GABAergic neurons (Fig. 5C and Supplementary Digital Content Figure S3B, available at: http://links.lww.com/JS9/F180). This suggests that MTX may selectively target specific cell types in the hippocampus, disrupting transcriptional regulatory networks in GABAergic neurons by inhibiting the sustained expression of key TFs, thereby leading to abnormal neuronal network activity. Subsequently, we identified 11, 17, and 17 significantly downregulated TFs in GABAergic_1, 2, and 3, respectively. Among these, 4 TFs were commonly downregulated in GABAergic neurons, 5 were specifically downregulated in GABAergic_1, and 8 were specifically downregulated in GABAergic_2 and GABAergic_3 (Fig. 5D). By constructing TF-target regulatory networks and performing GO functional pathway enrichment for the potential target genes of these cell-specific downregulated TFs, we found that nearly all of the significantly downregulated TFs were closely associated with nervous system development. In addition, TFs specifically downregulated in different GABAergic subtypes were involved in specific biological processes. For instance, TFs downregulated in GABAergic_1 and 2 may play important roles in synapse assembly, transmission, adhesion, and GABA signaling pathways, while those downregulated in GABAergic_3 were more involved in transcriptional regulation and post-translational modifications (Fig. 5E and Supplementary Digital Content Figure S3C, available at: http://links.lww.com/JS9/F180). Among these downregulated TFs, we focused on Etv5 in GABAergic_1, which is related to cell adhesion; Mbnl2 in GABAergic_2, associated with synaptic transmission; and Egr1 in GABAergic_3, involved in synaptic plasticity regulation (Fig. 5F). Therefore, the suppression of these TFs in GABAergic neurons may be a crucial factor contributing to cognitive deficits. These findings indicate that MTX-induced cognitive dysfunction is likely due to the dysregulation of transcriptional regulatory networks mediated by multiple functional TFs in GABAergic neurons.

### Cell trajectory analysis of neurons

**Figure 6.**
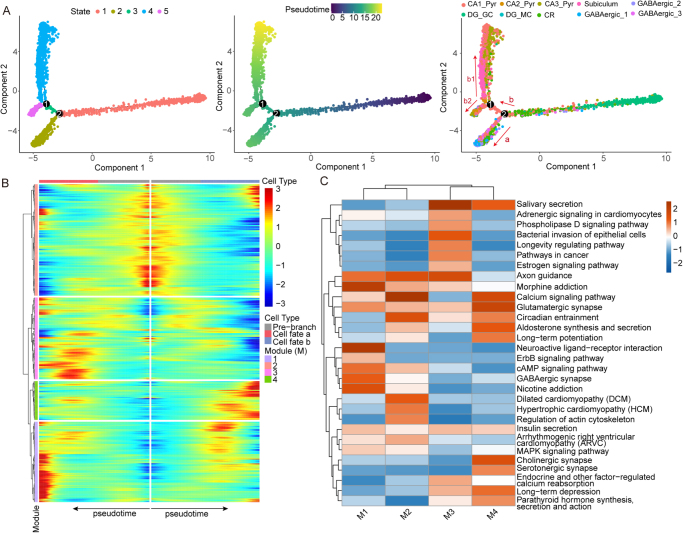
**Cell trajectory analysis of neurons.** (A) Pseudotime analysis results indicating, from left to right, cell states, pseudotime progression, and the distribution of different cell types. (B) BEAM analysis results at branch point 2. (C) Top 10 KEGG enrichment pathways for gene modules identified in the BEAM analysis.

To further clarify whether MTX treatment induces abnormal neuronal developmental trajectories, we performed pseudotime analysis using Monocle2 and identified five distinct cell states (**States 1–5**). By referencing neuronal circuits, we modeled the differentiation trajectories of neuronal subpopulations and inferred multiple divergent differentiation paths leading to distinct cell fates, which suggesting that MTX may affect neuronal health (Fig. 6A). At branch point 2, the developmental trajectories of GABAergic neurons and other cells diverged significantly, evolving along two completely different paths. Trajectory a defined final fate of most GABAergic neurons (Supplementary Digital Content Figure S4A, available at: http://links.lww.com/JS9/F180). Additionally, we observed that the proportion of State 2 cells in the MTX group (21.92%) was significantly higher than in the NC group (16.09%) (Supplementary Digital Content Figure S4B, available at: http://links.lww.com/JS9/F180), suggesting that MTX treatment likely accelerated the differentiation process of neuronal cells toward trajectory a, promoting GABAergic neuron development.

Subsequently, we performed BEAM analysis to explore the pseudotime genes potentially responsible for trajectory changes at different branch points. At branch point 2, based on the expression of these pseudotime genes, they were grouped into four main gene modules (M1–4). M2 genes were primarily expressed in anterior branch cells at branch point 2 (i.e., State 1), while M4 genes were mainly expressed in posterior branch cells that determined cell fate b (i.e., States 3–5). Only M1 and M3 module genes were associated with determining cell fate a (i.e., State 2) (Fig. 6B).

To determine the potential functions of these module genes, we conducted KEGG pathway analysis, which revealed that the pseudotime expression of different module genes plays an important role in determining cell fate. The results showed that M4 module genes were highly associated with excitatory synapses, such as glutamatergic and cholinergic synapses, while M1 module genes were related to inhibitory synapses, such as GABAergic synapses (Fig. 6C). Although M1 also exhibited some association with glutamatergic synapses, this is likely due to the presence of a small number of excitatory neurons in the State 2 population (Supplementary Digital Content Figure S4A, available at: http://links.lww.com/JS9/F180). In summary, these findings further indicate that GABAergic neurons are predominantly clustered in State 2 cells, while excitatory neurons, such as pyramidal and subicular neurons, are mainly distributed in States 3–5.

We noted that although both M1 and M3 play important roles in determining cell fate a, the contribution of M1 appears to be greater, as some of the genes still show persistently high expression at late pseudotimes, likely playing a crucial role in the developmental maturation of GABAergic neurons. Therefore, we followed the pathways significantly enriched by M1 genes, which include neuroactive L–R interaction, cAMP signaling pathway, and ErbB signaling pathway, among others, suggesting their central role in MTX-mediated cognitive dysfunction (Fig. 6C). On the other hand, we also found that branch point 1 may determine the differentiation fate of CA2_Pyr cells, which could represent a characteristic feature of the CA2_Pyr-mediated disynaptic circuit as opposed to the trisynaptic circuit. Therefore, we similarly performed BEAM analysis separately at branch point 1 and further subdivided trajectory b into trajectory b1, which differentiates toward State 4, and trajectory b2, which differentiates toward State 5. The pseudotemporal expression of M5 module genes determines the fate of State 5 cells, while M6 module genes determine the fate of State 4 cells (Supplementary Digital Content Figure S4C, available at: http://links.lww.com/JS9/F180). KEGG enrichment results for different module genes show that the reduced activity of the calcium signaling pathway is likely a key factor preventing CA2_Pyr cells from developing along trajectory b1 (Supplementary Digital Content Figure S4D, available at: http://links.lww.com/JS9/F180). Thus, the calcium signaling pathway may play a guiding role in the formation and adaptive modification of neural circuits. These findings suggest that MTX treatment may cause abnormal neuronal developmental trajectories, particularly the transition toward GABAergic neurons, with various dependent neural signaling pathways, such as neuron activity-related L–R interactions, likely contributing to this process.

### MTX interrupts neural signaling by blocking interactions between neurons

**Figure 7.**
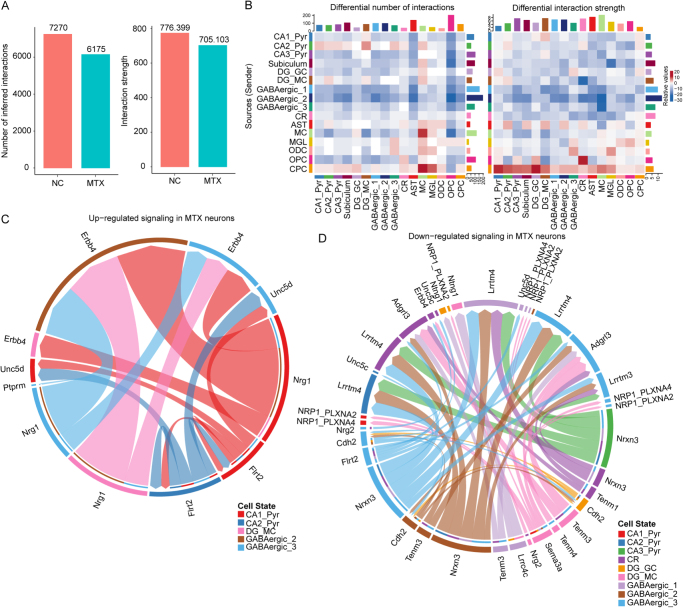
**MTX interrupts neural signaling by blocking interactions between neurons.** (A) Bar plot showing the overall number and interaction strength of intercellular communications between different cell groups. (B) Heatmap showing the differences in the number (left) and strength (right) of interactions sent and received by each cell type. (C) Circos plot showing upregulated ligand–receptor pairs in neuronal cells of the MTX group. (D) Circos plot showing downregulated ligand–receptor pairs in neuronal cells of the MTX group.

The interactions between neurons primarily depend on the transmission of neurotransmitters. Our results demonstrate that MTX directly disrupts the synthesis and release of neurotransmitters through its neurotoxic effects, effectively blocking interactions between neurons and interrupting neural signal transmission. To identify the potential cellular interactions and key signaling pathways affected by MTX, we analyzed and compared cellular communication among different hippocampal cell types using CellChat. Compared to the NC group, the overall number and strength of inferred intercellular interactions were significantly reduced in the MTX group (Fig. 7A). Furthermore, input and output strengths of neuronal cells (except OPCs) were consistently higher than those of glial cells (Supplementary Digital Content Figure S5A, available at: http://links.lww.com/JS9/F180), reflecting the possibility that OPCs may have strong communication capabilities, potentially forming direct synaptic connections with neurons and influencing neural network balance. By comparing the incoming and outgoing interaction signals of various cell types, we observed that MTX selectively weakened GABAergic neuronal interactions, while excitatory neurons remained relatively unaffected (Fig. 7B). This suggests that MTX may primarily inhibit the communication capacity of GABAergic cells.

Additionally, the number of interactions received by OPC cells from GABAergic cells in the MTX group was significantly reduced, reflecting the difficulty in transmitting GABA signals released from synapses. By comparing the proportions of information flow in key signaling pathways, we also identified some signal flows that were notably enhanced or diminished in the MTX group (Supplementary Digital Content Figure S5B, available at: http://links.lww.com/JS9/F180). Notably, we found that the Netrin signaling pathway (Ntn1–Dscam), which was significantly enhanced in the MTX group, is regulated by OPC cells (Supplementary Digital Content Figure S5C, available at: http://links.lww.com/JS9/F180). Ntn1 is considered an important axon guidance cue, acting as both an attractant and repellent, guiding GABAergic neurons expressing Dscam to migrate toward the substantia nigra reticulata. This directional migration may gradually exclude the migrating excitatory neurons from the reticulata and confine them to the compacta region. Thus, the Netrin signaling pathway may play a significant role in mediating interactions between OPC and GABAergic cells and maintaining the excitatory–inhibitory balance of neural networks.

Finally, we focused on cross-talk between neuronal subpopulations. Through L–R analysis, we found that the Nrg1–Erbb4 signaling pathway, from the excitatory neuron ligand Nrg1 to the GABAergic neuron receptor Erbb4, was significantly upregulated in the MTX group (Fig. 7C), suggesting that MTX may lead to excessive release of inhibitory neurotransmitters. Among the significantly downregulated pathways, the Nrxn3–Lrrtm4 signaling pathway caught our attention (Fig. 7D). qPCR analysis confirmed a robust reduction in Nrxn3–Lrrtm4 mRNA expression and an increase in Nrg1–Erbb4 expression in MTX-treated mice (Supplementary Digital Content Figure S6, available at: http://links.lww.com/JS9/F180). Moreover, MTX treatment significantly enhanced the fluorescence intensity of Nrg1–Erbb4 while markedly reducing the fluorescence intensity of Nrxn3–Lrrtm4 in the hippocampus of mice compared to controls (Supplementary Digital Content Figure S7, available at: http://links.lww.com/JS9/F180). Therefore, our findings indicate that the enhancement of the Nrg1–Erbb4 pathway and the weakening of the Nrxn3–Lrrtm4 pathway may disrupt the stability of trans-synaptic signal transmission between neurons, resulting in abnormal excitatory and inhibitory synaptic functions, ultimately manifesting as an imbalance in the brain’s excitatory/inhibitory neural network and leading to cognitive deficits.

These findings suggest that MTX exerts neurotoxic effects through modulation of interactions between neurons, and a deeper understanding of the synaptic transmission mechanisms underlying these interactions may help overcome or improve cognitive dysfunction induced by MTX.

## Discussion

In this study, we performed snRNA-seq on hippocampal tissue samples from MTX-treated and normal mice. Through cell type annotation and analysis of the proportions of different cell populations, we identified various neuronal and glial cells. MTX treatment reduced excitatory neurons and increased inhibitory neurons, along with elevated OPCs and decreased ODCs. These alterations may contribute to cognitive dysfunction by disrupting the excitatory–inhibitory balance within the neural network. Previous studies also found that humans who have undergone chemotherapy experienced a sustained depletion of oligodendrocyte lineage cells[43]. Consistent with these findings, and along with reduced central zone time, our mice also exhibited increased anxiety after chemotherapy exposure in open-field experiments. Using a mouse model of methotrexate-induced neurological dysfunction, we observed a similar reduction in white matter OPCs, accompanied by increased yet incomplete OPC differentiation and a lasting impairment in myelination. OPCs from chemotherapy-naïve mice similarly exhibit increased differentiation when transplanted into the microenvironment of previously methotrexate-exposed brains, indicating an underlying micro-environmental perturbation. MTX results in persistent activation of microglia and subsequent astrocyte activation, both of which are dependent on inflammatory microglia. Microglial depletion normalizes oligodendroglial lineage dynamics, myelin microstructure, and cognitive behavior after methotrexate chemotherapy. These findings indicate that MTX exposure was associated with persistent tri-glial dysregulation and identified inflammatory microglia as a therapeutic target to abrogate chemotherapy-induced neurological dysfunction. MTX induces prolonged microglial activation, which in turn triggers astrocyte activation, a process dependent on inflammatory microglia. Depletion of microglia restores normal oligodendroglial lineage dynamics, myelin microstructure, and cognitive function following MTX chemotherapy. Other studies also demonstrated that MTX reduces cortical Bdnf expression, which is restored when microglia are depleted. Bdnf–TrkB signaling is essential for activity-dependent myelination[44].

In chemotherapy-naïve mice, deleting TrkB specifically in OPCs leads to impaired cognitive performance. A small molecule TrkB agonist reverses both myelination deficits and cognitive impairments following MTX chemotherapy, but this recovery depends on intact OPC–TrkB expression. Our findings further indicated that synaptic activity in OPC from the MTX group is significantly enhanced, while glutamate signaling is markedly diminished, which may suggest a reduction in neuronal excitability accompanied by an enhancement of inhibitory actions, ultimately leading to a decline in neuronal activity.

Through neuronal subpopulation analysis, we identified several excitatory and inhibitory subtypes potentially associated with MTX treatment, including granule cells in the dentate gyrus (DG), hippocampal CA3 pyramidal neurons, and various GABAergic cells. These subpopulations may influence synaptic transmission and hippocampal circuit activity. Functionally, the CA1–CA3 regions contribute to memory processing (spatial, episodic, emotional) and information integration, while the DG—rich in granule and mossy cells—supports neurogenesis, spatial/contextual learning, and emotional regulation. Mossy cells, in particular, modulate both glutamatergic and GABAergic signaling, representing a potential therapeutic target for anxiety[45]. MTX may disrupt these circuits by altering neurotransmitter balance, potentially through changes in neuronal energy metabolism, ion homeostasis, or enzyme activity, thereby impacting excitatory–inhibitory transmission.

Furthermore, we identified cell-specific regulons in each neuronal subpopulation using the SCENIC tool, which include TFs and their target genes. Among these downregulated TFs, we specifically focused on Etv5 in GABAergic_1, which is associated with cell adhesion, Mbnl2 in GABAergic_2, which is involved in synaptic transmission, and Egr1 in GABAergic_3, which plays a role in regulating synaptic plasticity. Etv5 is essential for normal dendritic development in the hippocampus, and Etv5-deficient mice exhibit behavioral abnormalities and hippocampal connectivity defects, leading to cognitive impairment[46]. Similarly, Mbnl2 knockout mice display cognitive and emotional deficits[47], while Egr1 is considered a key mediator and regulator of synaptic plasticity and neuronal activity, playing a critical role in controlling cognition, emotional responses, and social behavior[48]. These results highlight potential therapeutic targets for further research into chemotherapy-induced cognitive impairment CICI.

In addition, we found that MTX induces abnormal developmental trajectories in GABAergic neurons. The differentiation trajectory of GABAergic cells (mainly GABAergic interneurons) is an important and complex research area in neuroscience. In the process of neural development, these cells go through a series of precise developmental steps and transformations from the embryonic stage, and eventually become cortical interneurons with different functions. In the later stages of development, the synapses of GABAergic interneurons undergo plastic changes, such as synaptic strengthening and synaptic pruning[49]. These processes are influenced by external environmental factors, neuronal activity, and synaptic modulators, ensuring the functional maturation of interneurons and the precise regulation of inhibitory control in the cerebral cortex. Abnormalities in the differentiation trajectory of GABAergic interneurons can lead to a range of neurodevelopmental disorders, such as autism[50], schizophrenia[51], and epilepsy[52]. These conditions are closely associated with impaired development of interneuron subtypes, migration defects, or abnormalities in synaptic connectivity. Our analysis provides potential therapeutic targets and mechanistic clues for MTX-induced cognitive impairment.

Finally, through intercellular L–R analysis, we found that the signaling pathway from excitatory neurons ligand Nrg1 to GABAergic neuronal receptor Erbb4 was significantly upregulated in the MTX group. Numerous studies have demonstrated that the Nrg1–Erbb4 signaling pathway plays a key role in mediating GABA release and some cognitive disorders, such as schizophrenia[53], epilepsy[54] and autism[55]. For instance, mutations in the Nrg1 or Erbb4 genes are linked to cognitive deficits commonly observed in schizophrenia, such as impairments in attention, working memory, and executive function. Abnormalities in this pathway may disrupt cortical signal transmission, thereby interfering with normal cognitive function.

Nrxn3 is a presynaptic membrane protein that plays a critical role in synapse formation and the maintenance of synaptic function[56]. Lrrtm4, an effective postsynaptic organizer, regulates glutamatergic synapse assembly and plays a critical role in excitatory synapse formation and presynaptic differentiation[57]. The Nrxn3–Lttrm4 pathway plays a crucial role in synapse development and neurotransmission, impacting both synaptic plasticity and cognitive function. Disruption of Nrxn3–Lrrtm4 interactions can reduce synaptic strength, compromise excitatory transmission, and impair synaptic plasticity—processes critical for long-term potentiation (LTP), learning, and memory. Aberrant Neurexin–Lrrtm4 signaling affects excitatory synapse formation, contributing to cognitive impairments like memory deficits and impaired executive function. Such changes can disturb the excitatory–inhibitory balance in neural circuits, a hallmark of disorders like schizophrenia.

Of course, there are some limitations to our study. Firstly, we recognize the need to apply additional methodologies to further elucidate the role of the Nrxn3–LRRTM4 signaling pathway in MTX-induced cognitive impairment. Secondly, our single-nuclei sequencing requires isolating individual cells from tissues or samples. For certain tissue types or cell populations, the process of isolating single cells can be challenging and may result in the loss of some cell groups, making it difficult to fully represent cellular diversity. Additionally, the number of single cells that can be sequenced in an experiment is limited, which makes it hard to comprehensively capture rare cell types or the diversity in hippocampal tissues. Another limitation of our study is the use of snRNA-seq, which captures only a subset of the full transcriptome. Nuclear RNA represents approximately 20% of the total cellular RNA, and key cytoplasmic transcripts, including those rapidly degraded or localized for translation, may not be detected in snRNA-seq. As a result, some functionally relevant genes, particularly those involved in dynamic processes like synaptic transmission or immediate early responses, might be underrepresented or missed altogether. While snRNA-seq offers advantages in preserving the spatial and cellular diversity of frozen tissue samples and enables access to cell-type-specific transcriptomes, caution is warranted when interpreting changes in pathways dependent on cytoplasmic or translational regulation. To mitigate this limitation, we complemented our analysis with pathway-level and transcription factor network analyses to infer broader regulatory trends. Nevertheless, further validation using full-length single-cell RNA-seq or spatial transcriptomics may help refine and confirm our findings. Future studies should incorporate in vivo functional validation using genetic approaches (e.g., conditional knockout or knockdown models) and pharmacological interventions (e.g., pathway-specific antagonists) to directly assess their contributions to MTX-induced neurotoxicity. In addition, longitudinal behavioral assessments and region-specific manipulations could provide a more comprehensive understanding of how these pathways modulate hippocampal circuitry and cognitive outcomes.

## Conclusion

In our experiment, we successfully established a mouse model of cognitive impairment induced by the chemotherapy drug MTX. Using snRNA-seq, we uncovered the complex impact of MTX on various cell types within the hippocampus of mice and revealed several cell populations and key functional signaling pathways that may be associated with cognitive impairment. Our findings suggest that dysregulation of the transcriptional regulatory network and impaired cell–cell communication among GABAergic neurons are closely linked to cognitive deficits. The reduced capacity for cellular interaction induced by MTX may impair the stability of intercellular synaptic signaling. Alterations in critical synaptic transmission pathways, such as Nrg1–Erbb4 and Nrxn3–Lrrtm4, appear to be key contributors to excitatory–inhibitory imbalance and cognitive dysfunction. These insights provide a crucial basis for developing new potential targets and therapeutic strategies.

## Data Availability

The data supporting the findings of this study are available from the corresponding author upon reasonable request.

## Research registration unique identifying number (UIN)

No human participants were involved, and therefore, no approval from a human research ethics committee was required.
